# Porcelain Inlays and Restorations

**Published:** 1903-07

**Authors:** W. T. Reeves

**Affiliations:** Chicago, Ill.


### International Dental Journal.

VOL. XXIV. JULY, 1903. NO. 7.

### Original Communications.[1]

### PORCELAIN INLAYS AND RESTORATIONS.[2]

BY W. T. REEVES, D.D.S., CHICAGO, ILL.

MR. PRESIDENT AND MEMBERS OF THE ACADEMY,—In presenting this paper for your consideration this evening, I have given it a broad title in order that all phases pertaining to the use of porcelain as a restorer of lost tooth-structure may properly be brought out in the discussion. The discussion is oftentimes a most valuable complement to a paper.

The word inlay has come down to us from the first users of porcelain as a filling-material, and still clings to us. Its use is criticised by the low-fusing contingent, who claim that they make fillings, and that the advocates of high-fusing porcelain make inlays only.

"Inlay" properly described the work in its first conception, because it was porcelain inlayed into tooth. "Filling" is no broader in describing what we are doing to-day with porcelain,

[1] The editor and publishers are not responsible for the views of authors of papers published in this department, nor for any claim to novelty, or otherwise, that may be made by them. No papers will be received for this department that have appeared in any other journal published in the country.

[2] Read before the Philadelphia Academy of Stomatology, January 27, 1903.

because we do more than fill,—we build up, restore to original form and contour. "Restoration" would seem to fully describe all cases from the simplest to the most extensive that we have to deal with.

We have several divisions or methods among porcelain workers. Some use high-fusing, others low-fusing bodies. Each of these classes of workers is divided again as to methods, but we are all agreed upon the many good qualities of porcelain as a filling-material. It would hardly seem necessary at this late day—in fact, it is not necessary—to say anything in defence of porcelain as a filling-material. Opposition that was very outspoken and in no uncertain terms two and three years ago is now very guarded, and is trying to ride along under the cloak of conservatism, admitting its usefulness and that it has a place in dentistry, "when indicated," leaving you to form your own judgment how much "when indicated" covers. Now, I have no quarrel with the conservative man. I believe, in most places and under most conditions, he is the safe man. It is only when he unintentionally takes the place of the "dog in the manger" that I want to enter a protest. It oftenest happens that the persons heard from under these conditions are those who are looked upon as leaders in the profession, and their negative endorsement "when indicated" is often the means of keeping many good men from taking up the work; for they say, and think, if Dr.'s X, Y, and Z give it but that scanty endorsement, they had best wait for further developments. I say of Dr.'s X, Y, and Z not that they are unknown, but that they are unknowing. Such discussion as, "I have made a few inlays for labial cavities and cavities that are not subjected to stress; such cavities in the anterior teeth where gold would be unsightly I believe inlays are indicated, etc., etc.," practically means that such person has made so few inlays as to know nothing about the technique of the work, consequently his judgment as to "where indicated" is not worth much. If he were one-tenth as well prepared to handle porcelain as a filling-material as he is to handle gold, he would go from easy to more difficult cavities, until there would practically be no limitations in his hands and he would be as warm an admirer of porcelain as the most enthusiastic of us. Again, how discouraging to the uninitiated or the beginner must be that oft-repeated remark, "Oh, yes, Dr.'s A, B, C, and D can do nice work; they have a special gift for the work, but the great majority of dentists will never succeed with porcelain inlays." I do not believe there is any gift about it. I believe that what one person can do, all can do. To whatever walk of life you turn, those who have become prominent in their several callings are those who were workers. The genius is like a "flash in the pan," he soon passes away, but the one who attains to and retains a prominent place among his fellow-men is the worker. It is only a matter of the different degrees of application necessary. How many instances can be cited in all walks of life where success has been achieved over what seemed insurmountable obstacles at the beginning. I do not want to seem to hold out that there are any obstacles for you to overcome in taking up porcelain work. I believe that if every one present to-night would give to mastering the technique of porcelain inlays but one-tenth of the time he gave to mastering gold as a filling-material, he would have fifty per cent. greater success with porcelain as a tooth-saver than he has with gold.

X, Y, and Z object to the length of time required to make a good inlay, stating that they could put in gold in half the time. With very few exceptions this is not true. Small pin-head cavities, fissures in bicuspids and molars, especially the fissure extending from the distal pit over on to the lingual surface of superior molars, can be quickly and successfully filled with gold. Aside from these few cases I know of no cavities that cannot be filled with porcelain in less time than they could have been filled with gold on the average. I know that to-day I am handling more patients and putting in more fillings with porcelain than I was able to do with gold. Any one would be foolish to undertake to perform any task with which he was but slightly familiar, or one that he performed so seldom as not to "have his hand in," if he expects to accomplish it in the same time as one who is doing that work daily. That applies with full force to the handling of porcelain as a filling-material. Who of you does not remember how long it took to put in gold fillings when you began your practice, fillings that you now put in better in one-third of the time simply through every-day familiarity with the material. If you will practise for six months, and then for the second six months will keep strict account, you will find that it takes less time with porcelain than with gold, with less physical strain upon both yourself and your patient. In the second year you will put in very few gold fillings.

Right here I want to enter a protest against certain statements that appeared in a recently published paper. In the January issue of the *Dental Cosmos* was published a paper by a Western man read before a State society in the far West. The preparation of an approximal cavity in a central incisor, involving a portion of the cutting edge, was described. I quote the paragraph following said description:

> "The preparation of such a cavity in most instances is to the experienced and artistic worker only a matter of five or ten minutes, the making of a matrix five or ten minutes more, and fifteen minutes for baking and dressing leaves ample time for cementing and discharging well within the hour."

Either the inlay made in that length of time is a very imperfect, slovenly piece of work and not worthy of being called a porcelain inlay, or the writer has not kept well within the bounds of truth. How discouraging this must be to the beginner! It takes me from two to three times that long to make a porcelain restoration for an approximal cavity involving a portion of the cutting edge. There is one thing that porcelain has no competition with, and that is time. Every step and detail of the work must be carefully executed. The least defect at any stage of the work means failure of the whole. The inlay must be perfect in fit and contour, or it should not be set. It may not be as good as desired in color, but if perfect in fit and contour it will do perfect service for the patient.

We have passed the experimental stage. Porcelain as a filling-material has come to stay. It will not answer any longer to put off inquiring patients with the stock remark that it is only a fad, that you do not advise their use, and that their particular case is one in which porcelain would be a failure. Fads develop quickly and pass as quickly. Porcelain inlays have been fifteen years and longer reaching their present stage of development, and have won their way against the most stubborn opposition that was ever waged against any new material or method brought before modern dentistry. The way has been blazed by pioneers in the work, and methods and materials have been developed to a high stage of completeness. It only remains for the profession at large to enter upon the work, and enter it under conditions that are favorable to success. One thing they must realize, and that is, that the mere getting of a furnace, an outfit of bodies, and a little platinum, and reading a few articles on the subject in the dental journals, is not all they will have to do to start them on porcelain inlay work. They must prepare themselves by good technique instructions, then careful, thorough dummy work in the laboratory. The work is not so difficult as to be a bugbear if one but enters the path from the right direction and passes along with a sure, steady step. There is a good deal of pessimism about the foregoing, and by nature I am an optimist, and cannot refrain from giving you a little of that optimism in connection with porcelain as a filling-material. I have prepared and read six papers before as many different State societies this past year on this subject, and while I have endeavored to make them individual, I have, from necessity, quoted more or less, particularly in describing the purely technical work. I am going to make this broad statement: There are scarcely any cavities in which gold can be utilized that porcelain cannot be successfully used, and there are so many places and conditions under which gold cannot be used and where porcelain will be a perfect material with which to restore lost tooth-structure that I place it first in point of applicability.

I will not take your time to enumerate all the places in which porcelain can be used, but only some cavities in which gold cannot be used and where porcelain will do perfect service, and then some in which porcelain is the only material indicated.

For the young, in whose mouths gold fillings fail faster than cement fillings wash out, and where cement washes out so fast as to be dangerous, one may come very near doing permanent work with porcelain.

For the aged, whose strength will not permit the long sittings necessary for the insertion of a gold filling, one can do permanent work with porcelain, because the work can be divided between two or three sittings.

For those bordering on nervous prostration, and those with high-strung nervous temperaments, for whom it is a physical impossibility to prepare a cavity, even for a cement filling, to say nothing of gold, one can do permanent work with porcelain. In these cases porcelain is a boon to both patient and operator, for there is no material we use for the reception of which the cavity can be prepared with as little discomfort to the patient as for the insertion of a porcelain inlay; there is no necessity for extension for prevention, or for undercuts.

For those refined, sensitive natures, to whom the display of gold in the front of the mouth is an ever-conscious annoyance, one can confer a lasting benefit by the use of porcelain, and restore teeth so that at close conversational range fillings would not be perceptible.

In teeth that are loose from pyorrhœa one can do permanent work with porcelain, whereas one could not pack gold either by hand-pressure or mallet, and would have to resort to amalgam or cement.

The other conditions, which are by far the most important, and which prove a quality that has not been credited to porcelain and bring out a point I want to make, are the following, in which porcelain only is indicated: In extensive cavities, in which decay has approached so closely upon the pulp that death of the pulp would be almost sure to follow if a metallic or cement filling be used, one may fill with porcelain with almost absolute certainty that the pulp will remain alive.

Cavities on the buccal or labial cervices of molars, bicuspids, or incisors that remain sensitive to thermal shocks when filled with gold become perfectly normal when gold is replaced by a porcelain inlay.

When, after loss by fracture of a large portion of an anterior tooth, it becomes desirable to retain the pulp alive, on account of incomplete development of the root, the lost portion may be restored to full contour and usefulness by a porcelain tip, and the pulp will remain alive.

In these and other similar cases, nature revolts at the introduction of a metallic or plastic material for the replacement of the lost tissue, but is perfectly resigned to the introduction of porcelain in the form of inlays. Compatibility of porcelain to tooth-structure is a quality belonging to porcelain that we have not apppreciated, because heretofore we have had no material that possessed this quality. Compatibility of material to tooth means a great deal; it means more than one realizes at first. If nature could reproduce lost tooth-structure, that would be ideal restoration. But nature being unable to do this, art steps in with porcelain, and in true art the artifice is hidden. The definition of compatible, as given by the Standard Dictionary, is, "1. Capable of existing together, congruous, consistent. 2. Being in harmony, mutually tolerant, accordant, congenial."

In our search after an ideal filling-material we have looked for one that would be easy of manipulation, permanent in preserving quality, and harmonious to its environment.

That we have in porcelain a material that possesses these qualities there is little doubt, and yet the possibilities of porcelain are as little appreciated to-day as were the possibilities of electricity twenty years ago.

Compatibility may be in part the explanation of the well-known clinical fact as to why there is seldom or never any recurrence of decay about a porcelain inlay or restoration. Scientific theories are to be respected in so far as they have been demonstrated, but in the face of practical demonstration or clinical facts scientific theories have to readjust themselves. You may say that all the materials with which we fill teeth are immune to caries. I grant this in the strict interpretation of the word, but with the exception of one they do not bring immunity to the mouth. Gold, either in the form of crowns or fillings, no matter how highly polished, will tarnish and collect increasing amounts of food deposits. All other materials will collect and retain food deposits, and thus be an ever-present menace to approximating tooth-structure.

*Glazed porcelain will not retain food deposits, and is not affected by the fluids of the mouth.*

I have always contended that an inlay should never be ground on any surface other than the occlusal surface in molars and bicuspids and on the cutting edge in the anterior teeth. It is the practice of a good many inlay workers, after setting an inlay, to grind the surface to make it level with the plane of the tooth, and they thus lose a large per cent. of the quality of immunity.

This is necessitated by the use of low-fusing bodies, which have a tendency to assume a spheroidal form in fusing, and in high-fusing bodies by over-building or over-contouring. All necessary grinding should be done before the matrix is removed and the inlay again glazed. This glazed surface is a protection to surrounding and approximating tooth-surfaces, and brings immunity to a greater extent than any other material or agent we use. The tooth that has for approximate contact point the glazed surface of porcelain is better protected by sixty per cent. from liability to decay than when both contact points are natural tooth-structure, while the tooth that has gold or other filling-materials for contact point is more endangered by twenty per cent. than in the original condition.

Before giving the technique of the work in detail, I want to say something of color selection. I want to bring before you a new principle in color selecting. It is the general practice to select the color as one selects a facing for a crown,—by taking the shade-guide furnished with every outfit of bodies and selecting the color that seems to be a match for the tooth. In selecting a facing general effect is aimed at and the selection is one that harmonizes with the adjoining teeth. A person may select a facing that, placed between two natural teeth, cannot be detected, but if a corner of that facing is joined to the corner of either adjoining tooth it will not match at all, because each tooth has different underlying colors. It is the underlying colors that must be looked for, and it is just in proportion as one is able to see these underlying colors, and then to reproduce them in the inlay, that he will be successful. A typical light-yellow tooth will probably have either a gray or a blue tinge, or both, in its inside substance. Those same colors should be reproduced in the inlay, or it will not be a perfect match. I use the shade-guide not to get a match for the tooth, but as an aid in looking for the underlying colors and the depth of those colors.

If you see yellow, brown, blue, gray, or all of them, hold the shade-guide to the tooth and determine the strength of these colors, also the order in which you will use them to get the effect you want. Make a memorandum of this. For instance, you desire a match to a typical light-yellow tooth; the cavity extends from neck to cutting-edge, involving at the cutting edge a third (more or less) of the width of the tooth. Yellow No. 2 on the Brewster shade-guide looks to be a perfect match for the tooth, but at the neck you will find the yellow a little deeper, with a grayish tinge through the centre third of the tooth and a bluish tinge towards the cutting edge; you will find on looking closer that the blue seems to reflect through a gray and that there is very little yellow in this part of the tooth. My memorandum would read thus: Patient A.—Foundation, cervical, No. 3 yellow; centre, No. 6 gray; cutting edge, No. 9 blue under No. 5 gray; over all, No. 2 yellow, with No. 11 for enamel.

I would proceed to use them as follows: Build my foundation; the yellow, gray, and blue would then be put in their respective places and baked as one layer; have the three bodies properly moistened and arranged on the slab so that you know which is which; take as nearly as you can judge the proper amount of each and place at their respective places; then draw a gnarled handle gently over the pliers to bring the moisture to the surface and cause them to run together; cease instantly, and you will have a perfect blending of the colors; if you continue to jar, you will get a mixture instead of a blend. Allow the body to dry, and bake.

Shrinkage will necessitate the addition of more of these colors, and this time, as you want gray over the blue, you will use only No. 3 yellow and No. 5 gray; have the bodies ready and proceed as before; this will still further harmonize your colors, because the yellow will extend still farther down your inlay and modify the gray that was put on in the central portion, while the blue of the lower third modifies the gray, so that when they are all covered with the lighter yellow you have such a harmonizing of colors that you can't tell it from nature's work itself.

If shrinkage causes any lack of contour, and it most always does, cover all with a layer of Brewster's XX body. This is a new product. You cannot say it is of any particular color unless you call it enamel color. So far it seems to fill its mission perfectly.

You now have a completed inlay that I believe comes the nearest to reproducing nature of any means or methods heretofore attempted. A still further artistic effect can be obtained by the use of what I call primary colors. They are as deep as ivory black, Prussian blue, Van Dyck brown, burnt ochre, etc. They are high-fusing bodies and not paints, but they must be used as paints would be, by putting them on mixed with oil, for you cannot put them on thin enough or smooth enough when mixed with water as you do ordinary bodies.

These are a set of colors that were made for me by Mr. Brewster, of Chicago, and are meeting a long-felt want.

With them you can imitate that steel-blue line you so often see just above the cutting edge, also tobacco stained teeth and the white and yellow mottled teeth. These effects have heretofore been impossible, but with the primary colors they can be reproduced with life-like naturalness.

THE TECHNIQUE OF PORCELAIN INLAYS AND RESTORATIONS.

I do not think I can do better than to detail my method of making inlays, and afterwards give you my reasons for such procedure.

It is almost needless to say that I am a strong advocate of high-fusing bodies. By that we mean bodies that fuse above the melting-point of gold. Of necessity, to use such bodies something fusing at a higher point than gold has to be used with which to form a matrix. Platinum $^1/_{1000}$ of an inch in thickness is the best and most desirable material for matrix formation.

Platinum $^1/_{1000}$ of an inch in thickness is what I have always used, and it has several qualities that make it superior to platinum $^1/_{2000}$ of an inch in thickness. The very fact that it requires more work and care to burnish into the cavity will cause a more perfect matrix to be made when the work is accomplished; also it will have more stability and reduce the danger of warpage, while baking the porcelain, to the minimum.

The burnishing of the matrix I divide into four divisions or steps; needless to say that the annealing and burnishing is repeated at each step. A piece of $^1/_{1000}$-inch platinum is cut large enough to extend well beyond all the margins other than the cervical margin. After annealing in the Bunsen flame, place in position so that the surplus will be about equally distributed beyond the margins of the cavity. Take a piece of wet cotton or spunk and place so as to depress the platinum into the cavity, using a minimum-sized round-nosed burnisher and adding additional pledgets of the wet cotton as the platinum is carried to place, care being taken that at no time are you in danger of the burnisher going through the cotton. At this step you do not attempt to turn the platinum over the margin. Remove the cotton and matrix from the cavity, and if your packing has been thorough you will find the platinum has been carried to every part of the cavity and your margins are fairly well defined, with little or no danger of breaking through the platinum. If you have broken through the platinum inside the cavity, no harm will result, as the body will bridge across without danger of flowing through. If you have any surplus at the cervical margin that will press upon or hurt the gum while burnishing, trim away at this time. Anneal and return to the cavity for the second step.

Pack partially full of wet cotton, but not over the margins; then, using any method that is easiest, turn the platinum over all the margins and down upon the tooth. To accomplish this I use a piece of ordinary twilled tape, passing it up so as to engage all portions of the platinum at once, then draw it in the direction that will cause the platinum to lay over the margins and down upon the tooth in all directions, thus bringing the folds equally well distributed, and fairly well burnished to the surface of the tooth.

Remove from the cavity, and if the platinum has lapped over the tooth far enough to bind and hold into the cavity, trim away until the matrix will remove from the cavity freely. Anneal and return to the cavity for the third step.

At this stage your work is entirely upon the margins. The platinum that lapped over on the tooth will enable you to hold the matrix into the cavity securely enough with your fingers for you to accomplish this work. All wrinkles or folds must be burnished out and beyond the margins in both directions. It matters not if the matrix springs from one part of the cavity while burnishing upon another, although you will endeavor to hold it firmly in place; the next step will correct all faults of that nature.

The burnisher should be used so that the head bears upon the margin cavityward and the shank upon the margin toothward; in this manner you will burnish every portion of the margins until you are satisfied with their adaptation to the tooth. Also burnish the interior of your cavity so that your matrix will have as close adaptation inside the cavity as at the margins.

It may be necessary for you to anneal several times before you complete this stage of the work, but do not leave this stage until it is thoroughly completed.

We now come to the last and final burnishing. I employ a method by which the matrix is held firmly to every part of the cavity and at the same time does not interfere with the burnishing of every part of the matrix. For that purpose I use a strip of rubber dam which is drawn tightly and binds the matrix into the cavity in all directions. You can now go over all the margins and surfaces and burnish out all the spring there may be in any part of the matrix. It is not necessary to spend as much time in burnishing at this stage as at the previous stage, as the close fit is already practically secured.

Now comes the task of removing the matrix from the cavity without warping it. The capillary attraction of the saliva between the matrix and the tooth will often hold it very tightly. By patient and careful teasing you can accomplish this without any warping. If sprung ever so slightly, dry the cavity and matrix and repeat the last burnishing. Grasp the edge of the platinum at some point as far removed from the margin as possible, and where there is no fold to make it rigid, with a pair of laboratory pliers that can be locked, burn off the saliva in the Bunsen flame to avoid any possibility of gassing. The matrix can now be handled freely in building in the porcelain, for the bending of the platinum will take place at the pliers and not near the margins. At this stage, if desirable, the patient can be dismissed, as there is no necessity of reburnishing any part of the matrix after having done a baking.

For illustration of handling the body, I will describe the making of an inlay for an approximal cavity in a central incisor extending from the cervical or gum margin to and including a third or half of the cutting edge. Select some one of the several good bodies that are on the market to build what I call the foundation of the inlay. I use close body or Brewster's foundation. Have it finely ground, for it will pack more solidly, carve better, and shrink less than the coarser ground. Build it into the matrix little by little, jarring it down well to make it solid; build out the corner in excess of what you expect the contour to be, to allow for shrinkage; when sufficiently dry, carve it to shape. Carve the lingual surface right down to the contour the matrix gives you, then carve away what will be the labial half of the inlay, so that the room for laying on your colors will be the same at the edge of the tooth as at any other part of the inlay. Bake this, and if you have estimated your shrinkage correctly you have practically an inlay for the lingual half of the cavity; if shrinkage has been more than expected, build on more of the foundation and bake again.

You are now ready to build on the colors you want the inlay to be, and you can vary the shading from neck of tooth to cutting edge at pleasure. I never mix two or more bodies together to vary the shade of one, but depend on the thickness of the layer to give me the shade I want. I can best illustrate this by showing the effect of holding a sheet of colored glass to the light; you get a certain shade of that color; now place another sheet back of it, and you get a deeper shade of the same color, and by adding more sheets of glass you get a still deeper shade of the same color. Build your colors on in layers and bake each layer as you go along. When you have built your colors to almost full contour cover the whole inlay with a neutral color that will allow the underlying colors to reflect through; in this way you will get a translucent effect, and it is the only way in which natural translucency can be obtained. In the tooth you are trying to match the colors are all in the dentine and reflect through the enamel; the enamel of all teeth is practically the same color.

All inlays, whether restoring contours or simple saucer-shape cavities, I build up in layers, and never less than three layers,—foundation, color, and enamel.

Before stripping the platinum from the inlay, try into the cavity to see if you have the contour perfect, also to judge of the occlusion as near as you can by observation. You cannot have the patient close upon the inlay, because previous to its being set it would be easy to fracture the porcelain. After the cement is completely crystallized a thin porcelain filling in the occlusal surfaces of molars will have the full strength of the whole tooth to resist masticating stress and is in no danger of fracture.

If the contour is lacking, build on more body; if too full, grind and change as desired, and return to the furnace to be glazed again.

I will now give my reasons for taking more time than the majority of inlay workers spend in baking an inlay.

*Why build in layers?* An inlay built up in layers accomplishes three objects,—first, a natural-looking, translucent inlay; second, an inlay built of three or more layers of different body will break up the absorption of light, so that from whatever angle or point of view you look at it, it will look practically the same, while an inlay built all of one body or mixture will absorb light all in one direction, and viewed from one point will look all right, but from the opposite point of view will show up as plain as black and white; third, that great bugbear of most inlay workers, the cement showing through after the inlay is set, is overcome. You will often hear operators say that they had a splendid color before the inlay was set, but after it was set the cement killed it entirely. That was because the inlay was all baked of one body and the cement could reflect through from underneath as easily as the light was absorbed only in one direction from above.

The three points I claim for this method are translucency, avoidance of shadow, and prevention of cement reflection from underneath.

I will close by giving you a law of physics that has not been understood or appreciated by inlay workers.

A dentist has been brought up all his life on the one law of self-retentive form of cavity and interlocking form of filling, and it is hard for him or inlay workers to break away from that law. There are inlay workers to-day who are working upon self-retentive forms of cavity formation, and grooving their inlays or baking into them platinum pins and loops to make them as near interlocking as possible.

I believe inlays depend for their strength of retention upon close adaptation and the crystallization of the cement under pressure. If two sheets of glass are adapted, with water between them to exclude the air, one cannot forcibly pull them apart. A joiner prepares the surfaces of two pieces of wood so that they are in close adaptation to each other, and placing glue between them clamps them into a vice and leaves them to harden. If there be any appreciable amount of glue, there will be no strength of joint. There is no strength in the glue. The strength is due to close adaptation and crystallization under pressure.

If this be true, there is no need of inflicting the additional pain upon the patient that the self-retentive style of cavity formation would entail.

I hope I may have given you some ideas and principles that, if studied and followed, will lead to a larger and more extended use of porcelain as a filling-material.

